# Experience of tinnitus in adults who have severe-to-profound hearing loss: A scoping review

**DOI:** 10.3389/fneur.2022.1004059

**Published:** 2022-10-28

**Authors:** Lama Alzahrani, Magdalena Sereda, Carla Salles Chamouton, Háula Haider, Rebecca Susan Dewey, Derek J. Hoare

**Affiliations:** ^1^NIHR Nottingham Biomedical Research Centre, Nottingham, United Kingdom; ^2^Hearing Sciences, Mental Health and Clinical Neurosciences, School of Medicine, University of Nottingham, Nottingham, United Kingdom; ^3^Audiology Clinic, Otolaryngology Department, King Abdul-Aziz University Hospital, Jeddah, Saudi Arabia; ^4^UNICAMP University of Campinas, Campinas, Brazil; ^5^ENT Department, Hospital Cuf Tejo—Nova Medical School, Lisbon, Portugal; ^6^Sir Peter Mansfield Imaging Centre, School of Physics and Astronomy, University of Nottingham, Nottingham, United Kingdom

**Keywords:** tinnitus, deafness, priority question, assessment, experience

## Abstract

**Background:**

Tinnitus is defined as the subjective perception of sound in the absence of an external stimulus, and tinnitus disorder becomes relevant when it is associated with emotional distress, cognitive dysfunction, and/or autonomic arousal. Hearing loss is recognized as the main risk factor for the pathogenesis of tinnitus. However, clinical guidelines for tinnitus disorder provide little direction for those who have severe-to-profound hearing loss including those who are pre-lingually Deaf. The aim of this scoping review was to catalogue what is known from the existing literature regarding the experience and management of tinnitus in adults who have a severe-to-profound hearing loss.

**Summary:**

A scoping review was conducted following the Preferred Reporting Item for Systematic Reviews and Meta-analysis extension for Scoping Reviews. Records were included if they reported an evaluation of tinnitus in adults who had severe-to-profound hearing loss. The online databases Ovid (MEDLINE, EMBASE and PsycINFO), CINAHL, ProQuest, Scopus, and Google Scholar were searched using the search terms ‘tinnitus’ (as a MESH term) and ‘deaf’ OR ‘profound hearing loss. Thirty-five records met the inclusion criteria for this review and were cataloged according to three major themes: Impact of tinnitus in deaf adults; Primary treatment of tinnitus in deaf adults; and Cochlear implant studies where tinnitus was a secondary outcome. Tinnitus symptom severity was assessed before and after intervention using tinnitus validated questionnaires in 29 records, with six further records using other assessment tools to measure tinnitus severity. Participants using cochlear implants were included in 30 studies. Medication, repetitive transcranial magnetic stimulation (rTMS), electrical promontory stimulation, and behavioral self-control therapy were each reported in single records.

**Key messages:**

This scoping review cataloged the experience, assessment, and treatment of tinnitus in adults who have severe-to-profound hearing loss. It is shown that there is very limited research reported in this field. Although this review included many records, most focused on the provision of cochlear implants for severe-to-profound hearing loss, with assessment and measurement of tinnitus as a baseline or secondary outcome. Largely missing in the literature are empirical studies that seek firstly to understand the nature of the experience of tinnitus by people with no or little residual access to external sound.

## Introduction

Tinnitus is the conscious awareness of a tonal or composite noise for which there is no identifiable corresponding external acoustic source. This becomes tinnitus disorder “when associated with emotional distress, cognitive dysfunction, and/or autonomic arousal, leading to behavioral changes and functional disability” (1). An estimated 10–15% of the adult population experience tinnitus, and around 1–2% of all people are severely affected (2). Hearing loss is considered a significant risk factor for tinnitus (3).

In terms of clinical guidelines, little reference is made to the management of tinnitus in those who have severe-to-profound hearing loss beyond the management of hearing loss with hearing aids or cochlear implants (4). Indeed, the UK National Institute for Health and Care excellence (NICE) tinnitus guidance specifies research in this area as being of high priority, e.g., there were no standardized assessments or questionnaires that could be used to make evidence-based recommendations for adults with severe-to-profound hearing loss (5). Furthermore, many tinnitus studies and clinical trials focus on participants who have less severe hearing loss sufficient for them to have good access to common sound-based or talking therapies for tinnitus (6, 7).

The aim of this scoping review was to broadly understand the state-of the art in this field by cataloging research to date that has included participants who had severe-to-profound hearing loss and tinnitus.

## Materials and methods

This scoping review was conducted and is reported according to the Preferred Reporting Items for Systematic reviews and Meta-Analyses extension for Scoping Reviews (PRISMA-ScR) guideline (8).

### Eligibility

The inclusion criteria were based on the PCC (Population/Concept/Context) mnemonic (9) (Table 1).

**Table 1:** Inclusion and exclusion criteria.

**Criteria for inclusion**	**Criteria for exclusion**
Adults aged 18 years and older	Children
Bilateral severe/profound hearing loss or	Adults with normal or mild-to-moderate
Deafness	Hearing loss
Subjective tinnitus	Objective tinnitus
Chronic tinnitus (>6months)	Sudden or unilateral hearing loss
Published in English language	Not in English

*Target population:* adults aged 18 years or older, bilateral severe-to-profound pre- or post-lingual hearing loss/deafness who also report subjective tinnitus lasting more than 6 months.

*Concept:* Experience of chronic subjective tinnitus in individuals with severe-to-profound hearing loss. Experiences could include accounts of personal life experiences, and management strategies included but were not limited to clinical assessments, education, counseling, and sound-based therapies such as hearing aids or cochlear implants given prior and after intervention for comparsion.

*Context*: no restrictions regarding time or geography.

Studies were excluded if they only included adults with normal hearing or mild-to-moderate hearing loss with no reference to severe-to-profound hearing loss. Studies of objective tinnitus, studies involving children, and studies not available in English were also excluded.

### Information source

To identify potentially relevant records, the following databases were initially searched in July 2020 and updated in February 2022: MEDLINE, EMBASE, PsycInfo, Web of science, CINAHL, ProQuest, Scopus, EThOS, Pubmed, and Google Scholar. The search strategies were drafted and refined by the review team through discussion and time frame was open. Search results were exported into EndNote, and duplicates were removed.

### Search strategy

Medical Subject Heading terms of tinnitus, hearing loss, and were searched as keywords (example search strategy for MEDLINE as in Table 2).

**Table 2:** An example search strategy used for MEDLINE *via* Ovid SP.

1	Tinnitus/
2	Limit one to (English language and “all adult (19 plus years)”)
3	Phantom sound.mp.,
4	Limit three to (English language and “all adult (19 plus years)”)
5	Tinnitus in old age or tinnitus intensity or “tinnitus is the perception of sound in the absence of auditory stimulation for 36 of the population it seriously interferes with many aspects of life a trauma focused approach is hypothesized to reduce tinnitus distress treatment with EMDR showed significant results persisted for up to 3 months in follow up” or tinnitus patients or tinnitus problems or tinnitus related distress or tinnitus related fear or tinnitus sensitization or tinnitus severity or tinnitus sufferers. kw.
6	Two or four or
7	Hearing loss/ or hearing loss, sensorineural/ or deafness/
8	Limit seven to (English language and “all adult (19 plus years)”)
9	Profound hearing loss.mp.
10	(Profound hearing loss or profound no syndromic hearing impairment or profound sensorineural hearing loss or profound SNHL or profound deafness or profound hearing impairment).kw.
11	Eight or nine or 10
12	Adult/
13	Geriatrics/
14	Twelve or 13
15	Six and 11 and 14

### Selection of source of evidence

Two reviewers (LA, DH) screened all records first by titles and abstracts, and if either reviewer considered the record potentially relevant or if insufficient information was provided to decide it was progressed to full text screening. Records were included if both reviewers considered them eligible. Where disagreement arose, the record was discussed with a third reviewer (MS) and consensus taken to include or not.

### Data charting process

Data were charted in Excel Supplemental Information 2 according to the aim of the scoping review. The Excel form was piloted using five records and revised before formal extraction started. Two reviewers (DH, LA) extracted data independently. Extracted data were compared and revisited if required to agree a single final dataset from each included record.

### Data items

Data items extracted were as follows: publication year, study design, country, population description including description of hearing loss, etiology of hearing loss, impact of tinnitus, comorbidity, assessment tools used to assess tinnitus or comorbidities, intervention, effects of intervention, and any other relevant finding and recommendations.

### Synthesis of results

Extracted data were discussed among researchers (DH, LA, MS) to explore different options for grouping the data according to themes. Data were grouped according to three major themes: ***Impact of tinnitus in deaf adults**, **Primary treatment of tinnitus in deaf adults***, and ***Cochlear implant studies where tinnitus was a secondary outcome***.

### Expert consultation

After data synthesis a draft manuscript was shared with three experts in the field (Speech and language therapist, ENT consultant, deafness researcher) (CSC, HH, RSD) with substantial practical experience in tinnitus and hearing loss. They provided a review of the manuscript and in particular a critique of the reviewer interpretation that had been placed on the dataset, and the relevance of the review to current clinical need and practice. Feedback was incorporated into the manuscript in an iterative manner.

## Results

**Figure 1:**
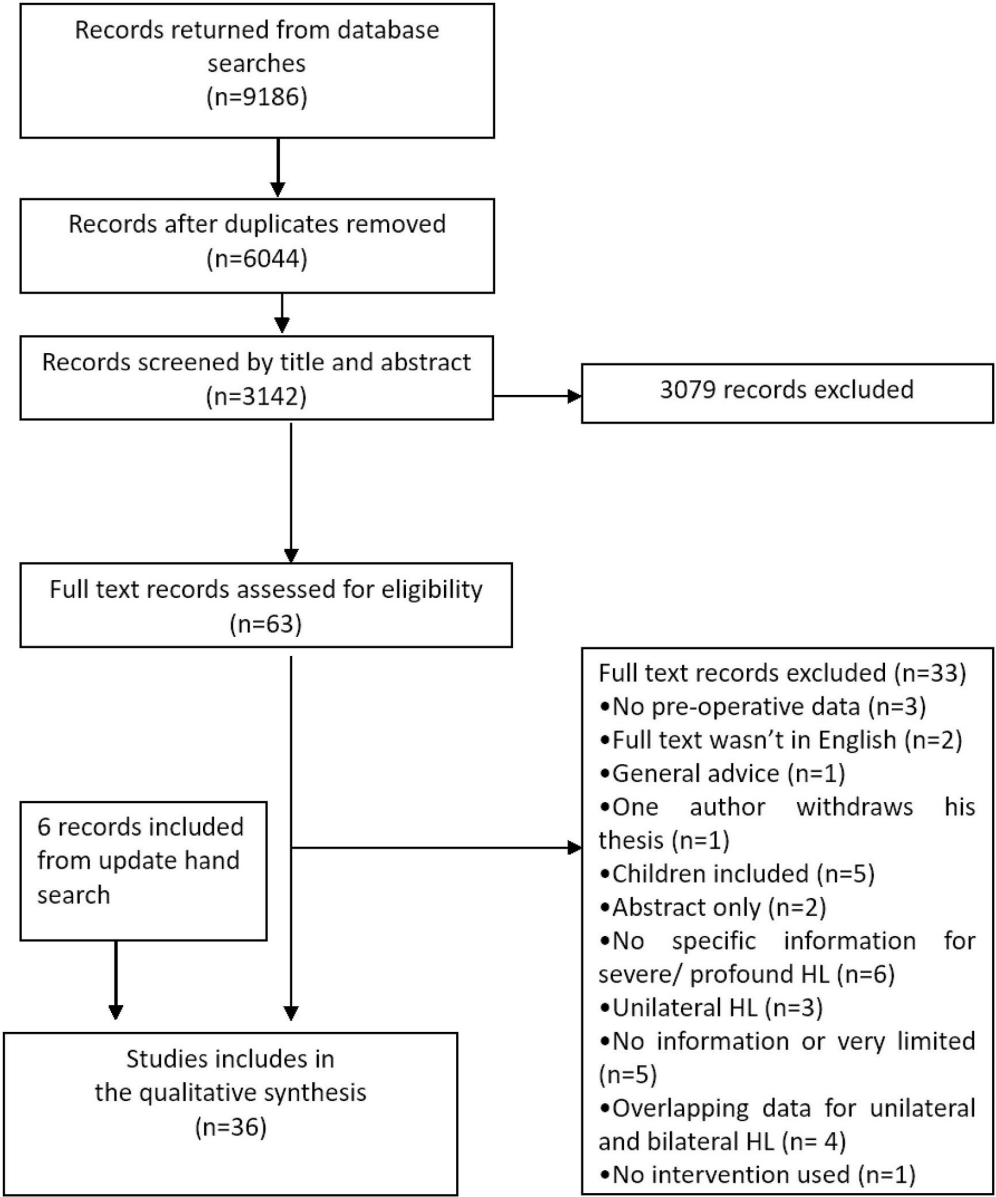
Flowchart showed the process of extracting, screening and analyzing the data.

Searches returned 9,186 records of which 6,044 duplicates were removed. Hence, the abstract and title of 3,142 records were screened for potential inclusion. The result was 63 records eligible for full text screening. After full text screening 36 records were included (Figure 1). Reviewers only disagreed on one record for inclusion/exclusion, and in this case the record was discussed with a third reviewer leading to a majority decision to include the record.

### Study demographic

**Figure 2:**
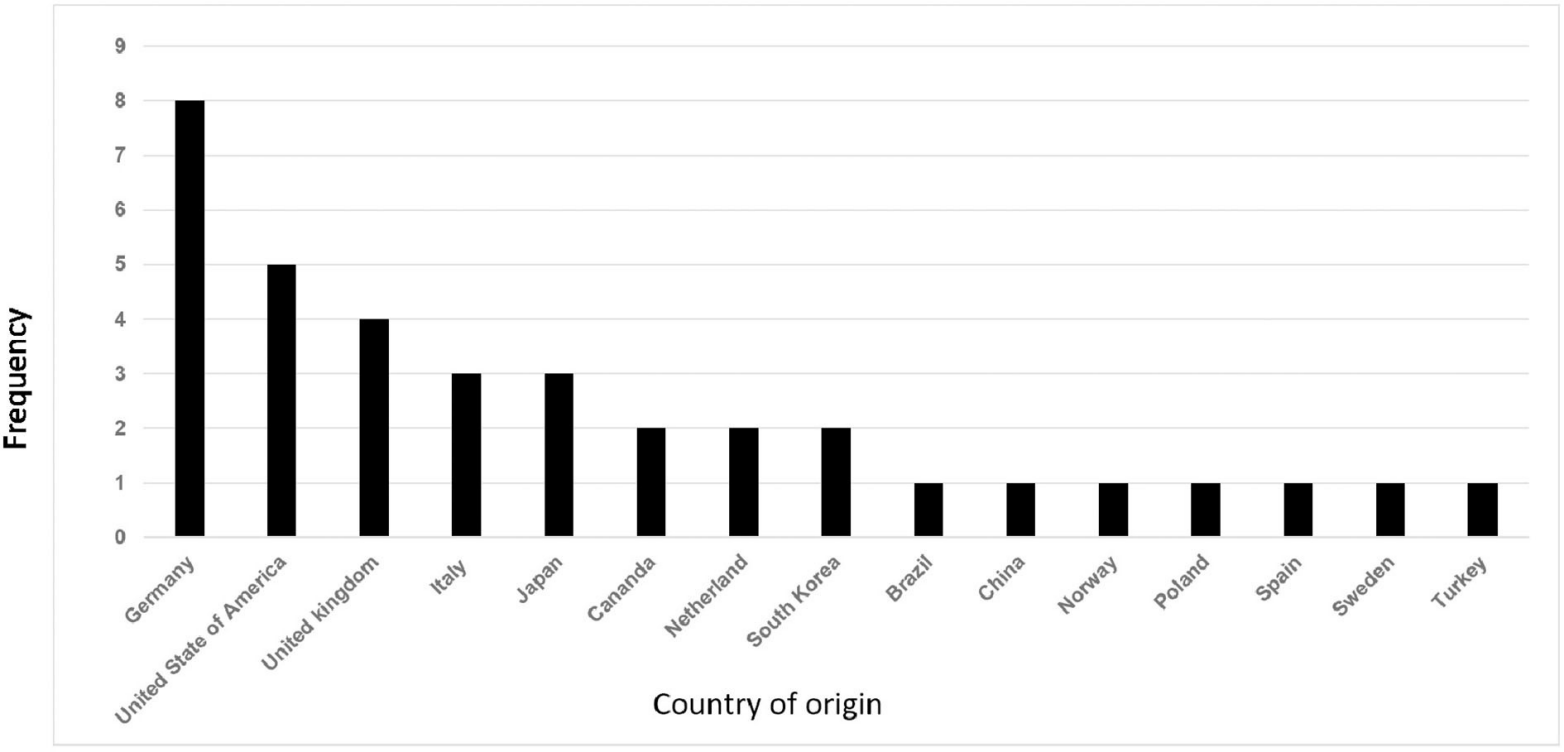
Distribution of included studies by country of origin.

**Figure 3:**
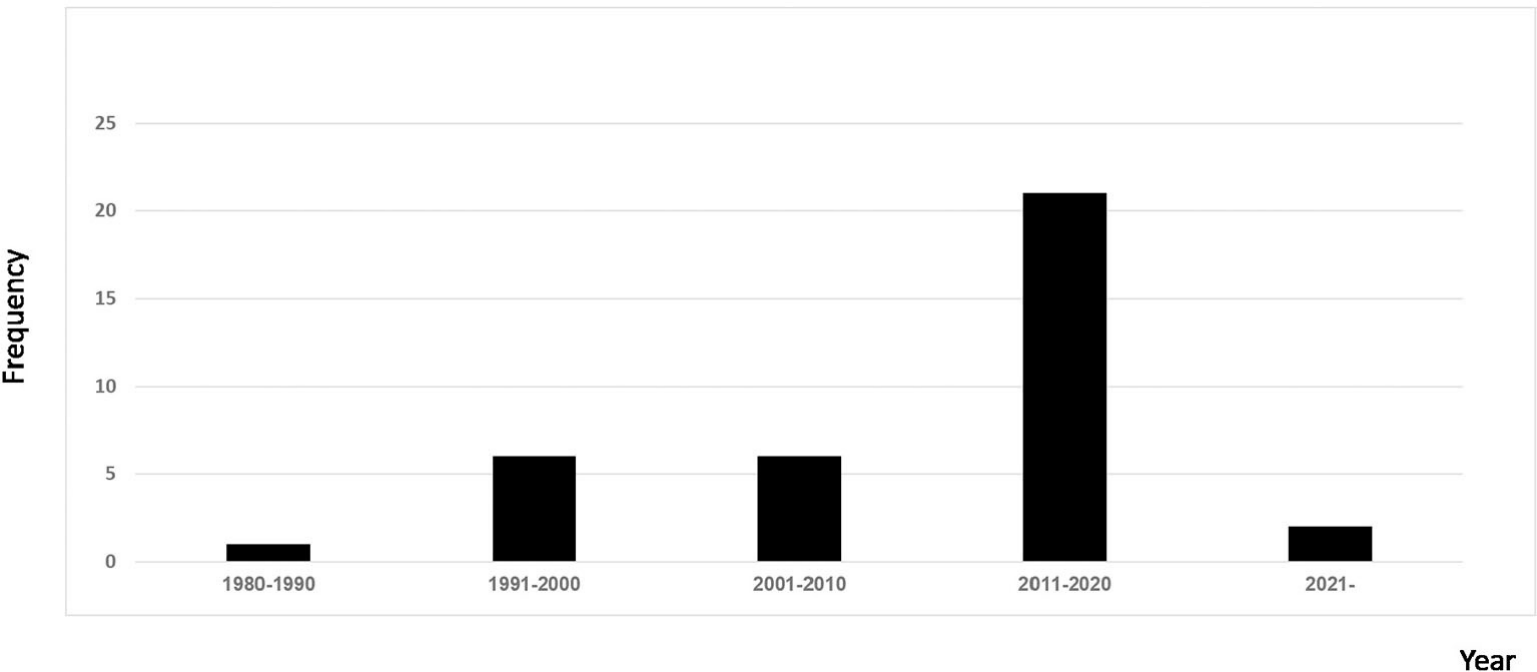
Distribution of included studies by year.

Of the 36 included records (Table 3) (10–23), 14 were prospective case studies, 11 were retrospective case reviews, three were case reports, two were cross-sectional studies, two were part of randomized controlled trails, one was a narrative, one was a case study, one was a non-randomized controlled clinical trial, and one was a combined retrospective and prospective study. The earliest record was published in 1988 and the most recent was published in 2021. Records originated from 15 countries with most studies originating from Germany (Figure 2). Most studies were published in the last decade (Figure 3).

**Table 3:** Characteristics of included studies (year, author(s), title, country, study design, study population, size of sample with tinnitus, and age).

	**Year**	**Lead author**	**Title**	**Country**	**Study design**	**Study population**	**Total sample (with tinnitus)**	**Age**
1	1988	Lindberg Per	Effects of self-control training on tinnitus in a deaf patient: A case study	Sweden	Case study.	Post-lingually deafened	1 (1)	26
2	1992	Charles R. Souliere,	Tinnitus suppression following cochlear implantation. A multifactorial investigation	USA	Retrospectivee cohort study.	Cochlear implant users (severe-profound SNHL)	33 (28)	21–74
3	1994	Juichi Ito	Tinnitus suppression by electrical stimulation of the cochlear wall and by cochlear implantation	Japan	Retrospective case study	Cochlear implant users with pre implant tinnitus (severe-profound SNHL)	30 (26)	18–63
4	1994	Juichi Ito	Suppression of tinnitus by cochlear implantation	Japan	Retrospective case study.	Cochlear implant users with pre implant tinnitus (severe–profound SNHL)	20 (18)	8–61
5	1995	Richard S. Tyler	Tinnitus in the profoundly hearing-impaired and the effects of cochlear implants”	USA	Retrospective cohort study	Profoundly deaf cochlear implant users	82 (22)	34–68
6	1997	Richard T. Miyamoto	Electrical suppression of tinnitus *via* cochlear implants”	USA	Retrospective cohort study.	Cochlear implant users (severe-profound SNHL)	64 (49)	Range 3rd−8th decade
7	1998	Y. Fukuda	The AllHear cochlear implant and tinnitus	Brazil	Case report	Cochlear implant users with pre-implant tinnitus (severe-profound SNHL)	6 (6)	17–64
8	2001	Michael J. Ruckenstein	Tinnitus suppression in patients with cochlear implants	USA	Prospective cohort study	Cochlear implant candidates who complain of tinnitus pre-implantation (severe-profound SNHL)	38 (38)	>18
9	2007	Walter Di Nardo	Tinnitus modifications after cochlear implantation	Italy	Retrospective case studies	Cochlear implant users (severe-profound SNHL)	30 (20)	16–74
10	2008	Nicola Quaranta	The effect of unilateral multichannel cochlear implant on bilaterally perceived tinnitus	Italy	Prospective cohort study	Cochlear implant users who complain of tinnitus pre-implantation (severe-profound SNHL)	62 (41)	17–77
11	2009	Tao Pan	Change in the Tinnitus Handicap Questionnaire After Cochlear Implantation	Canada	Retrospective cohort study	Cochlear implant users who complain of tinnitus pre-implantation (severe-profound SNHL)	244 (153)	18–90
12	2009	Walter Di Nardo	Transtympanic electrical stimulation for immediate and long-term tinnitus suppression”	Italy	Prospective cohort study	Post-lingual monaural or binaural profound hearing loss and with severe and disabling tinnitus in the worse ear	11 (11)	34–64
13	2010	Elisabeth Masgoret Palau	Tinnitus and cochlear implantation. Preliminary experience	Spain	Retrospective cohort study/ Case Reports	Cochlear implant users who complain pre implantation of disabling tinnitus (severe-profound SNHL)	3 (3)	32–57
14	2011	Heidi Olze	Cochlear Implantation has a Positive Influence on Quality of Life, Tinnitus, and Psychological Comorbidity	Germany	Prospective cohort study	Cochlear implant users with tinnitus pre-implantation (severe-profound SNHL)	43 (39)	19–77
15	2012	Heidi Olze (a)	Extra benefit of a second cochlear implant with respect to health-related quality of life and tinnitus	Germany	prospective cohort study	Cochlear implant users (severe-profound SNHL)	40 (28)	Not reported
16	2012	Heidi Olze (b)	Elderly patients benefit from cochlear implantation regarding auditory rehabilitation, quality of life, tinnitus, and stress	Germany	prospective cohort study	Cochlear implant users (severe-profound SNHL)	55 (20) older 55 (35) youngers	17–67
17	2012	Heidi Olze (c)	The impact of cochlear implantation on tinnitus, stress and quality of life in post-lingually deafened patients	Germany	prospective cohort study	Cochlear implant users who complain of tinnitus pre-implantation (severe-profound SNHL)	32 (28)	19–77
18	2013	Dong-Kee Kim	Tinnitus in patients with profound hearing loss and the effect of cochlear implantation	South Korea	Retrospective cohort study	Cochlear implant users (severe-profound SNHL)	35 (22)	47.5 ± 15.1
19	2015	David Greenberg	Developing an assessment approach for perceptual changes to tinnitus sound characteristics for adult cochlear implant recipients	United Kingdom	Prospective cohort study	Cochlear implant users with tinnitus preimplantation (severe profound SNHL)	68 (64)	31–68
20	2015	Ingo Todt	Relationship between intracochlear electrode position and tinnitus in cochlear implantees	Germany	Retrospective cohort study	Cochlear implant users with tinnitus pre-implantation (severe-profound SNHL)	55 (36)	Not reported
21	2015	Sarah M. Theodoroff	Experimental Use of Transcranial Magnetic Stimulation (TMS) to Treat Tinnitus in a Deaf Patient	United states of America	Case study	Prelingually deaf	1 (1)	26
22	2015	Wheeler, S. L.	Tinnitus: A Deaf hearing Phenomenon	United Kingdom	Narrative	Prelingually deaf (Waardenburg)	1 (1)	Not reported
23	2016	Alice van Z	Effect of unilateral and simultaneous bilateral cochlear implantation on tinnitus: A Prospective Study	Netherlands	Prospective cohort study (part of randomized controlled trails)	Cochlear implant users with tinnitus pre-implantation (severe-profound SNHL)	38 (16)	Not reported
24	2016	Dong-Kee Kim	Prospective, Multicenter Study on Tinnitus Changes after Cochlear Implantation	Republic of Korea	Prospective cohort study	Bilaterally-deaf cochlear implant candidates (severe-profound SNHL)	79 (59)	51.5 ± 14.7
25	2016	Robert H. Pierzycki	Tinnitus and Sleep Difficulties After Cochlear Implantation	United Kingdom	A population-based cohort, prospective	Cochlear implant candidates who complain of tinnitus and did not receive implant (severe-profound SNHL)	211 (113)	40–69
26	2016	Steffen Knopke,	Impact of cochlear implantation on quality of life and mental comorbidity in patients aged 80 years”	Germany	prospective cohort study	Cochlear implant candidates (severe-profound SNHL)	17 (12)	<80
27	2016	Ying Liu	Suppression of Tinnitus in Chinese Patients Receiving Regular Cochlear Implant Programming	China	Prospective study, randomized controlled	Cochlear implant candidates who complain of tinnitus pre-implantation (severe-profound SNHL)	234 (108)	>18
28	2017	Piotr H. Skarzynski	Tinnitus severity in patients with cochlear implants”	Poland	interventional (experimental) clinical trials”	Bilateral cochlear implant users who complain of tinnitus pre-implantation (severe-profound SNHL)	46 (46)	18–85
29	2017	Steffen Knopke	Cochlear implantation of bilaterally deafened patients with tinnitus induces sustained decrease of tinnitus-related distress	Germany	prospective, longitudinal analyses	Bilateral cochlear implant users with tinnitus pre-implantation (severe-profound SNHL)	41 (41)	25-81
30	2018	Geerte G J Ramakers	Development and internal validation of a multivariable prediction model for tinnitus recovery following unilateral cochlear implantation: a cross-sectional retrospective study”	Netherlands	Retrospective cross-sectional study	Bilaterally-deaf cochlear implant users with tinnitus pre-implantation (severe-profound SNHL)	137 (87) Recovered *n* = 35 Non recovered *n* = 52	58.3–71.2 51.7–66.2
31	2018	Saeko Matsuzaki	Severe tinnitus in a patient with acquired deafness for over 50 years: A case report”	Japan	Case report	Deaf woman	1 (1)	68
32	2020	Manuel Christoph Ketterer	Binaural Hearing Rehabilitation Improves Speech Perception, Quality of Life, Tinnitus Distress, and Psychological Comorbidities”	Germany	Prospective cohort study	Cochlear implant users (severe-profound SNHL)	53 (29)	28–80
33	2020	Peter R. Dixon	Predicting Reduced Tinnitus Burden After Cochlear Implantation in Adults	Canada	Retrospective cohort study	Cochlear implant users with tinnitus pre-implantation (severe-profound SNHL)	358 (358)	≥18
34	2020	Elif Tugba Sarac	Effects of Cochlear Implantation on Tinnitus and Depression	Turkey	**Retrospective** cohort study.	Cochlear implant users who complain of tinnitus pre-implantation (severe-profound SNHL)	23 (23)	20–67
35	2021	Robert H. Pierzycki	Insomnia, Anxiety and Depression in Adult Cochlear Implant Users with Tinnitus	United Kingdom	Cross sectional	Cochlear implant users with tinnitus pre-implantation (severe-profound SNHL)	127(67)	Average age is (53.93) 18.98%
36	2021	Arne K. Rødvik	Sustained reduction of tinnitus several years after sequential cochlear implantation	Norway	Combined retrospective and prospective	Cochlear implant users with sequential bilateral CI for annoying tinnitus	20 (20)	Age range is 23.0–72.5 years

The characteristics of included studies are summarized in Table 3.

### Study population

Most records focused on post-lingually deaf cochlear implant users or candidates who reported tinnitus before implantation. Causes of hearing loss, where mentioned, included infections such as meningitis, or head trauma, and were described in some as either sudden or progressive. Few records reported on pre-lingually deaf adults.

#### Theme 1: Impact of tinnitus in D/deaf adults

Table 4 summarizes the assessment tools and evaluation of impact of tinnitus pre- and post-intervention, changes in tinnitus attributed to interventions and assessed comorbidities in D/deaf adults. Tinnitus assessment tools reported in the literature included validated tinnitus questionnaires (*n* = 26), in-house tinnitus questionnaires (*n* = 4), visual analog scales of tinnitus loudness or annoyance (*n* = 6), and minimum masking levels (*n* = 2). The validated tinnitus questionnaires used comprised: Tinnitus Handicap Inventory [THI; (24)] (*n* = 17), Tinnitus Questionnaire [TQ; (25)] (*n* = 7), Tinnitus Functional Index [TFI; (26)] (*n* = 2), Tinnitus Handicap Questionnaire [THQ; (27)] (*n* = 2), and the mini-Tinnitus Questionnaires 12 [miniTQ12; (28)] (*n* = 1). No problems with the administration of these questionnaires were reported and several had been translated into additional languages from the original. There were three studies which used in-house questionnaires containing questions about tinnitus duration, loudness, severity, and related comorbidities.

**Table 4:** Interventions used to assess and treat tinnitus.

**Re**	**Year**	**Author(s)**	**Tools used to assess tinnitus**	**Tinnitus impact pre-intervention**	**Intervention**	**Tinnitus impact post intervention**	**Comorbidities assessed**
1	1988	Lindberg, Per	Visual analog scale (VAS) for (STL) and (DT) and (TC)	Right sided tinnitus (high pitched) and changes all over the days and associated with headache and muscle pain	Behavioral approach	Reduced STL and DT and increase TC over time. She felt self-control and her associated symptoms improved.	None
2	1992	Charles R. Souliere,	Closed set of questionnaires assessing tinnitus loudness, location, and residual inhibition	Pre-operative loudness (6.5 ± 2.2) annoyance (5.6 ± 2.8)	Unilateral cochlear implant	(1) Loudness: 15 (54%) reported a tinnitus loudness decrease, 12 patients (43%) reported no change, and one patient (3%) noted an increase in loudness. (2) annoyance: postoperative annoyance (3.4 ± 2.9, 1 = 3.12, P < 0.002) Two patients (43%) noted a decrease in annoyance, 14 noted no change, and two (7%) noted an increase in annoyance.	None
3	1994	Juichi Ito	Tinnitus loudness was assessed either (marked, slight or none)	Tinnitus loudness was marked in eight cases (27%) and slight in 18 cases (13%).	Unilateral cochlear implant	Tinnitus disappeared: six cases (23%) Suppressed: 12 cases (46%) No change eight cases (31%) aggravated zero (0%) so tinnitus was abolished in 69%	None
4	1994	Juichi Ito	The degree of tinnitus is classified to marked, slight and none at time of promontory stimulation and after cochlear implant surgery	Prior to cochlear implant surgery, five patients (25%) had marked tinnitus and 13 patients (65%) had slight tinnitus.	Unilateral cochlear implant	At time of promontory stimulation: Four cases (22%) disappeared, and nine cases (50%) suppressed and five cases (28%) no change to their tinnitus. The degree of tinnitus changed after cochlear implant: Eight cases disappeared (44%), seven cases suppressed (39%), no change in two cases (11%) and aggravated in one case (6%) so it was disappeared or suppressed in 83 %	None
5	1995	Richard S. Tyler	THQ	Bothersome tinnitus and THQ overall score averaged 33.2% (SD = 24.7; range 8–84)	Unilateral cochlear implant	Mean total THQ was 31.2	Depression
6	1997	Richard T. Miyamoto	THI	Not reported	Unilateral cochlear implant	Mean THI post implant was 20.05	None
7	1998	Y. Fukuda	Not applicable	Six cases: (1) bilateral, high-frequency tinnitus of moderate intensity. (2) high-frequency disabling tinnitus in the head	Unilateral cochlear implant	(1) Tinnitus was relieved bilaterally. When the external unit is turned off, he has a residual inhibition of tinnitus for 10 min. (2)Tinnitus was relieved partially, with no residual inhibition.	None
				(3) A hissing-type bilateral tinnitus of mild in- tensity (4) high-pitched tinnitus of mild intensity (5) bilateral ringing tinnitus of moderate intensity. (6) She had no tinnitus before surgery.		(3) Tinnitus disappeared on both sides when the cochlear implant was turned on. She had residual inhibition of her tinnitus for 30 minutes. (4) Tinnitus was unchanged with the cochlear device. (5) Tinnitus disappeared in the ear in which the device was implanted. (6) Shock, pain, and tinnitus as soon as the electrical device was turned on.	
8	2001	Michael J. Ruckenstein,	A semiquantitative scale before and after cochlear implantation. Tinnitus is categorized based on its severity using a numeric scale	Twenty patients (55%) had marked tinnitus, described as severe or debilitating (level 4 or 5) scale 5: 15 cases (39.4%) scale 4: 6 cases (15%) scale 3: 13 cases (34.2%) scale 2: 4 cases (10.5%) scale 1: 0 cases	Unilateral cochlear implant	17 patients (45%) had a complete suppression of their tinnitus. Nine- teen patients (50%) had some suppression of tinnitus, and only three patients (5%) noted no change in their tinnitus levels. Thus, 35 of 38 patients (92%) noted a reduction in their tinnitus levels after implantation. No patient suffered an exacerbation of his or her tinnitus after implantation. Scale 5: 1 case (2.6%) scale 4: no scale 3: 5 cases (13.15%) scale 2: 15 cases (39%) scale 1: 17 cases (44.7)	None
9	2007	Walter Di Nardo	THI, tinnitus loudness, type of sound and its duration and severity.	Overall, THI scores were 44.5	Unilateral cochlear implant	Overall, THI scores were 22.75	Sleeping difficulties
10	2008	Nicola Quaranta	THI	The average THI score before cochlear implantation was 32 (standard deviation (SD) 24)	Unilateral cochlear implant	The average THI score after cochlear implantation was 12 (SD = 20)	None
11	2009	Tao Pan	THQ	THQ pre implant total score is 41.2, SD 22.35	Unilateral cochlear implant	THQ post implant total score is 29.8, SD 19.45	None
12	2009	Walter Di Nardo	THI and tinnitus pitch, loudness. Also, minimum masking level (MML) on the day of the EPS session	Total THI pre-EPS 49	Electrical promontory stimulation (EPS)	THI 1 month after EPS 33	None
13	2010	Elisabeth Masgoret Palau	THI	Case one THI was not measured. Case 2 THI = 94 Case 3 THI = 46	Unilateral cochlear implant	Case 2: THI = 70 at 6 months. Case 3 THI =10 at 6 months	Hyperacusis, and hypoacusis
14	2011	Heidi Olze	TQ	TQ scores were 30.9 -+ 18.8	Unilateral cochlear implant	TQ decreased to 23.6 6 15.8 after CI (P < 0.01)	Depression, stress and anxiety
15	2012	Heidi Olze (a)	TQ	Initial TQ score was 32.6 +/– 21.2.	Bilateral cochlear implant/ sequential within 6 months	TQ score decreased to 12.8 +/−12.5	Not assessed
16	2012	Heidi Olze (b)	TQ	TQ score of the older patients was 26.3 +/−23.1 where in younger patients TQ score before implantation was 29.1 +/–	Unilateral cochlear implant	TQ scores in elderly was 22.3 +/−17.7 While in younger group decreased 21.0 +/−15.3	Stress
17	2012	Heidi Olze (c)	TQ	TQ mean total score was 33.4	Unilateral cochlear implant	TQ mean total score was 20.3	Stress
18	2013	Dong-Kee Kim	THI	THI mean scores were 50.5+_28.7	Unilateral cochlear implant	THI mean scores were 10.1 ± 15.8	None
19	2015	David Greenberg	THI	Mean THI pre was 42 (moderate handicap)	Unilateral cochlear implant	Mean THI at 12 months was 22	None
20	2015	Ingo Todt	mini TQ12	Group 1: mean TQ12 6.9 Group 2: mean TQ12 3.8 Group 3: mean TQ12 4.8 Group 4: mean TQ12 6.7	Unilateral cochlear implant	Group 1: mean TQ12 6.3 Group 2: mean TQ12 2.5 Group 3: mean TQ12 5.4 Group 4: mean TQ12 9	None
21	2015	Sarah M. Theodoroff	TFI as a primary outcome measure and THI	TFI score 27.6 THI score 18	Repetitive Trans-cranial magnetic stimulation (rTMS)	TFI post TMS 44 and follow up after 26 weeks was 25.2 THI post TMS 18 and follow up after 26 weeks 14	Depression, anxiety
22	2015	Wheeler, S. L.	None	“Sometimes it's like a bomb—boom! Then my eyes swirl round as if I have spun round the room but I haven't and it has happened in a split second. Then a long wheeee whistle, winding down. then to a ringing ring, noise. I prefer the boom type because it slows down the tinnitus rather than the ringing one which goes on forever.”	No intervention/ proposed coping strategies	His brain get used to it.	None
23	2016	Alice van Zon	THI and TQ	Overall median THI score in unilateral pre op was 8 (2–32) where in bilateral it was 22 (0–48) Overall TQ score in unilateral 7 (0–33) while in was 20 (1–41) in bilateral CI	Unilateral and bilateral cochlear implant	Overall median THI score in unilateral post implant was 2 (0–6) where in bilateral it was 12 (0–28) Overall TQ score in unilateral 7 (0–21) while in was 9 (0–26) in bilateral CI	None
24	2016	Dong-Kee Kim	THI Korean version	THI mean total score pre implant was 45.5 ± 26.8	Unilateral cochlear implant	THI mean total scores immediately after implant was 40 and after 6 months 23	Depression and stress
25	2016	Robert H. Pierzycki	Self-reported measures of hearing, tinnitus type, and sleep difficulty in cochlear implant candidates in	Not reported	Unilateral cochlear implant	Not reported	Sleeping difficulties
26	2016	Steffen Knopke,	TQ	The mean value of TQ score before implantation was 18.5 +/-23.0	Unilateral cochlear implant	The mean value of TQ score post implantation was 13.2 +/−15.9	Depression Stress Anxiety
27	2016	Ying Liu	THI	Pre implant, mean THI control group 84 where mean THI programming group 80	Unilateral cochlear implant	**At 6 weeks:** Programming, 65 Control 70 **At 8 weeks:** Programming, 50 Control 64.5 **At 12 weeks:** programming 50 control group 60	None
28	2017	Piotr H. Skarzynski	THI andTFI	mean THI score pre-operative of the tinnitus was 39.9 Mean TFI 38.4	Unilateral cochlear implant	mean THI score pre-operative of the tinnitus was 25.6 Mean TFI 29.2	None
29	2017	Steffen Knopke	TQ	Mean TQ score 35	Unilateral cochlear implant	Mean TQ score 27.54	None
30	2018	Geerte G J Ramakers,	Self-developed questionnaires assessing tinnitus severity (mild/moderate/severe)	Not reported	Unilateral cochlear implant	Tinnitus recovery was evident in 40% while worsening of tinnitus following cochlear implant was 10% in years.	Anxiety Depression
31	2018	Saeko Matsuzaki	THI	THI score was at first visit 94	Medication (Antidepressant, sleep induction). Psychotherapy	THI score at 4.5 years was 0	Depression, anxiety
32	2020	Manuel Christoph Ketterer	TQ	Mean TQ pre-operative was 25.2	Bilateral sequential CI	Mean TQ post-operative in 24 months was 15.1	Anxiety Depression and stress
33	2020	Peter R. Dixon	THI	Mean THI score was 22 (0–50)	Unilateral cochlear implant	Tinnitus Handicap Inventory (THI) (reduction by at least seven-points) was observed in 262 (73.2%) patients, of whom 155 (59.2%) reported complete resolution.	None
34	2020	Elif Tugba Sarac	THI Turkish version	THI mean SCORE was 61 ± 26.2	Unilateral cochlear implant	THI mean SCORE was 36.9 ± 29.2	Depression
35	2021	Robert H. Pierzycki	THI	Mean THI in tinnitus group was 21.14	Unilateral cochlear implant	Not reported	Anxiety Depression Insomnia
36	2021	Arne K. Rødvik	THI	Mean THI score pre-implantation was 61.3	Bilateral sequential cochlear implant	Mean THI score post-second implantation was 20.3 (SD = 16.3),	None

THI, Tinnitus Handicap Inventory; TQ, Tinnitus Questionnaire; THQ, Tinnitus Handicap Questionnaire; TFI, Tinnitus Functional Index; mini TQ12, mini–Tinnitus Questionnaire 12; VAS, Visual Analog Scale; STL, Subjective Tinnitus Loudness; DT, discomfort from tinnitus, TC, ability to control tinnitus; SQ, Perceived Stress Questionnaire; BDI-Π, Depression Inventory II;BDI, Beck's Depression Index; STAI, State Trait Anxiety Inventory; GAD-7, Generalized Anxiety Disorder; HRQoL, Health related quality of life; MML, Minimum Masking Level.

The THI was used to assess tinnitus severity before cochlear implantation in 17 studies and once in a case study involving a Deaf female receiving antidepressant treatment. Although the TFI was used as a primary outcome measure to evaluate treatment including TMS and in unilateral cochlear implantation, it was generally used in conjunction with the THI.

Records commonly reported tinnitus experience either as a testimony from patients or in the format of the patient rating their tinnitus either using validated questionnaires or other assessment tools, e.g., Ruckenstein (29) assessed tinnitus severity using a semi-quantitative scale from 1= no tinnitus to 5= debilitating tinnitus. Some records recounted the individual experience: “*Sometimes it's like a bomb—boom! Then my eyes swirl round as if I have spun round the room, but I haven't, and it has happened in a split second. Then a long wheeee whistle, winding down. Then to a ringing ring, noise.”* This statement was from an adult who had been Deaf since birth describing his tinnitus attacks (30). He further stated that he preferred one type of tinnitus characteristic over others because of the duration “*I prefer the boom type because it slows down the tinnitus rather than the ringing one which goes on forever.”* Another statement from a 26-year-old deaf female with a hearing impairment attributed to an acoustic neuroma and right sided tinnitus (31) described her tinnitus as high pitched and a screaming sound that could go to an unbearable level several times a day. In general tinnitus characteristics such as type of sound, tinnitus duration, and localization were reported (32, 33).

#### Theme 2: Primary treatment of tinnitus in D/deaf adults

Included studies are described here according to whether the intervention was used primarily to treat tinnitus or to treat other conditions. There were only four studies where the study intervention was given primarily to treat tinnitus, and one conversation piece on coping strategies proposed by a Deaf patient. The latter was a case study of a 26-year-old female with deafness following removal of an acoustic neuroma, reporting the use of a behavioral treatment approach aiming to relieve tinnitus over five consecutive months with self-control muscular relaxation techniques resulting in an improvement following therapy whereby the patient reported that she felt in control of her condition (31).

Repetitive transcranial magnetic stimulation (rTMS) was used in one study where a 28-year-old post-lingually deaf (hearing impairment occurred after 5 years of age) female with tinnitus. The treatment consisted of 10 sessions of rTMS using 2,000 pulses/session and the stimulation rate of 1 Hz *via* a coil that was in adjustable stand against the left side of her head (34). Her tinnitus was not improved based on TFI and THI questionnaires scores.

A 69-year-old Deaf (hearing impairment occurred before 5 years of age) female complaining of severe tinnitus, as well as depression and anxiety since tinnitus onset (35). In this case, oral antidepressants (the selective serotonin reuptake inhibitor paroxetine hydrochloride, Paxil^®^, 12.5 mg, starting at one tablet a day and increasing to three tablets a day) was given. She additionally received night sedation (suvorexant, Belsomra^®^, 15 mg, one tablet a day). Her tinnitus was intermittent and subsided completely after 4 and a half years.

One record investigated tinnitus suppression following electrical promontory trans-tympanic stimulation in 11 patients with monaural or binaural profound hearing loss (36). Stimulations were given at various frequencies (50, 100, 200, 400, 800, and 1,600 Hz) at ascending levels to find the participant's threshold for at least 60 seconds and then the discomfort level in μA. Nine out of 11 patients (81.8%) had immediate suppression of their tinnitus following electrical promontory trans-tympanic stimulation with no worsening of tinnitus reported. The most effective stimulation frequencies were 50 and 100 Hz. However, data were pooled so the effects specific to bilateral hearing loss could not be extracted.

One study reported a conversation between two relatives. Both presented with Waardenburg Syndrome, one being Deaf since birth and the other exhibiting an unspecified hearing loss (30). They complained of different attacks of tinnitus with different descriptors such as bomb, whistle, and ringing. However, where the hearing participant sought medical advice for her tinnitus, the Deaf participant had never sought medical advice but adapted to ignore their tinnitus and to live with it. He also acknowledged the potential benefits of sound therapy, but this was not accessible to him due to his deafness. The hearing cousin found it shocking to discover that Deaf people can also experience tinnitus.

#### Theme 3: Cochlear implant studies where tinnitus was a secondary outcome

A cochlear implant was the most reported treatment (primarily for deafness) investigated in participants with deafness and tinnitus (Table 4). One record proposed a tinnitus recovery model following unilateral cochlear implantation in severe-to-profound hearing-impaired adults complaining of tinnitus based on several factors (37). Lower pre-operative Consonant-Vowel-Consonant (CVC) score, unilateral localization of tinnitus, and larger deterioration of residual hearing at 250 Hz were determined to be predictors of tinnitus recovery. Age at surgery and gender were also reported. Tinnitus recovery was reported in 40% (35/87) of included participants.

One study examined tinnitus suppression according to the method of electrode insertion during cochlear implant surgery (38). Participants were grouped into four groups according to route of electrode insertion whereby group 1: through a scalar change of the position of the cochlear implant electrode from scala tympani to the scala vestibuli; group 2: through perimodiolar electrode insertion in a scala tympani position; group 3: electrode was inserted *via* scala tympani; and group 4: electrode inserted *via* scala vestibuli due to obstruction of scala tympani (meningitis, otosclerosis). They observed tinnitus suppression in 73.6% of those in group 1, 50% in group 2, 60% in group 3, and 87.5% in group 4.

Three records considered tinnitus suppression over time following cochlear implantation. While two records found evidence for tinnitus suppression after 1 or 2 years respectively (39, 40). Kim et al. (41) significantly found tinnitus suppression 1 month and early period use of cochlear implant.

Three records (19, 42, 43) studied the impact of age, especially older age, on tinnitus in cochlear implants recipients. Olze (19) found that younger patients (age range 19–67) experienced greater suppression following cochlear implantation (pre-implant TQ scores was 29.1 while post implant decreased significantly to 21.0), older patients (age range 70–84) also had a reduction in TQ score (pre-implant TQ was 26.3 while post implant decreased to 22.3) but this improvement was not clinically meaningful. The second record reported that the prevalence of tinnitus was higher in the older age group (>40 years) than the younger group (<40 years). However, suppression of tinnitus was reported post-implantation in both groups with no new tinnitus being reported in those who did not have tinnitus pre-implantation (42). Finally, Knope et al. (43) found that tinnitus and psychiatric comorbidities were both improved post-implantation in elderly patients over 80 years old (mean TQ pre implant was 18.5 and decreased to 13.2 post implant, which represented a clinically meaningful improvement).

Bilateral sequential cochlear implant was examined in four records (21, 44–46) and reported as beneficial for tinnitus. However, newly induced tinnitus was also reported following implantation (in five out of 10 participants) in the simultaneous bilateral cochlear implantation (44). Von Zan (44) compared unilateral cochlear implant and bilateral cochlear implant in patients who complained of tinnitus pre-operatively. Sixteen patients were included in their trial (seven received unilateral and nine received bilateral cochlear implants). Tinnitus improvement was measured as change on THI and TQ scores. Scores on both questionnaires were significantly decreased over the post-implant in both unilateral and bilateral cochlear implant patients. However, a few cases of the newly induced tinnitus in patients who did not report preoperative tinnitus (five out of 10 in bilateral and one out of 12 in unilateral group) were also reported.

Olze (21) evaluated tinnitus suppression following bilateral cochlear implantation and found that participants who did not benefit from unilateral cochlear implant improved after their second implant. One record (46) provided sequential bilateral cochlear implants for annoying tinnitus in the un-implanted ear. THI and VAS Loudness and Annoyance were measured before the second sequential cochlear implant and 2 years after implant (short term) and 7 years post implant (long term). THI scores significantly decreased from 61. Three pre implant to 29.3 after first implant (SD = 23.5) and then 20.3 (SD = 16.3) post second implant.

One record examined introduction of the regular cochlear implant programing as a factor in tinnitus suppression (47). A sample of 108 patients with pre-operative tinnitus who received one cochlear implant was divided into a control group (*n* = 54) with no regular programming and a programming group (*n* = 54). The programing group had regular programming at weeks 6, 8, and 12 post-implants after switching on at week 4 whereas the control group had no regular programing post-activation. Both groups had decreased tinnitus handicap scores on THI, however improvement was slower in the control group (Table 4).

Finally, two records specifically investigated changes in tinnitus characteristics following cochlear implantation. Greenberg et al. (33) found that tinnitus was suppressed totally or partially in the ear ipsilateral to cochlear implant in 57% and in the ear contralateral to cochlear implant in 43% of patients when the processor was turned on. Further, Greenberg et al. (33) reported that humming was the most commonly experienced tinnitus sound by severe-to-profound hearing impaired individuals pre-implantation (68%) and the frequency of those reporting humming reduced (to 50%) post implantation. Conversely, Di Nardo et al. (32) found buzzing to be the most reported sound post implantation, followed by whistling, airplane/ship engine, and bells ringing. Di Nardo et al. (32) found that in a group of individuals pre-implantation, a single sound was present in 13 cases (65%) and multiple different sounds were reported in seven cases (35%). Post implantation, a single sound became the majority, being reported in nine cases (45%) and multiple different sounds were present only in three cases (15%).

## Recommendations for future research in the included studies

Authors of the included studies made various recommendations for further research, mostly related to the treatment of tinnitus. Theodoroff, SM and Folmer, RL (34) recommended that future studies of rTMS should be conducted to include more patients who have severe or profound hearing loss but who did not want to use hearing rehabilitation devices such as hearing aids or cochlear implants. Further studies on intracochlear stimulation and electrode insertion specifically to explore its effectiveness in tinnitus suppression and generation of new tinnitus (38) and programming parameters in cochlear implant recipients with tinnitus (48) were also recommended. Pan (49) recommended obtaining estimates of the magnitude of the tinnitus pre-implantation and whether tinnitus burden can be related to hearing improvement post-implantation. Laterality was also recommended to be considered in future studies to differentiate the effects of the cochlear implant surgery and cochlear implant activation on tinnitus perception. Finally, exploring the impact of specific symptoms, such as clinically significant insomnia, on the severity of tinnitus in cochlear implant recipients was also recommended, as were prospective studies to investigate insomnia, depression, and anxiety, and to adequately characterize and assess the clinical importance of any residual tinnitus-related symptoms after implantation (50).

## Discussion

This scoping review catalogs two key elements: firstly, the tinnitus experience of Deaf and severe-to-profoundly hearing-impaired adults. Secondly, the assessments and treatments that are offered or have been evaluated in the literature, many of which concern cochlear implantation.

### Tinnitus experience/ impact

Studies used various assessment tools including validated questionnaires which according to NICE guidelines (5) are necessary to assess the impact of tinnitus on patient and guide health care providers toward better management strategies. However, none of the questionnaires reported in the literature have been validated for use in Deaf populations (5). We don't know therefore whether they sufficiently capture the real impact of tinnitus, or the relevant changes in tinnitus severity for this population.

Validation studies should explore how pre-existing tinnitus questionnaire scores in a D/deaf population should be interpreted or develop and validate customized questionnaires or other measures of tinnitus severity and treatment-related changes in this population.

### Primary treatment of tinnitus in deaf adults

There were four records primarily concerned with the treatment of tinnitus in Deaf and severe-to-profound hearing-impaired adults, reporting four different treatment approaches, with variable outcomes. One study reported a case involving treatment of tinnitus with medication for comorbid depression and sleep deprivation. There is therefore no evidence to support medication use primarily for tinnitus (51).

One study reported the use of rTMS which is hypothesized to modulate neuronal activity over a large region of the brain using magnetic fields. This approach has been used extensively in small-scale studies of tinnitus with mixed evidence for its immediate effectiveness (52, 53) and little data on long-term safety, all authors proposing further and larger studies of this treatment approach. As such to date there is a strong recommendation against the routine clinical use of this method, which includes in those who are both deaf and have tinnitus (4).

A behavioral approach in another case study proved effective in alleviating tinnitus distress (31). The effectiveness of the Cognitive Behavioral Therapy (CBT) was evidenced for tinnitus management in people with less severe hearing loss, but its effectiveness has not been proven in deaf populations (4). Therefore, practice guidelines make no recommendation for CBT in Deaf and severe-to-profound hearing-impaired populations and those with limited conversational ability, and recent systematic reviews make no reference to the use of CBT in deaf patients (54, 55). Given the proven benefits of CBT for tinnitus studies, any necessary adaptations, and the trialing of CBT in deaf populations would be welcomed.

Electrical ear stimulation for tinnitus was used in one record with comparison to a cochlear implant. Although there was improvement in tinnitus using both approaches, improvements were greater for the cochlear implant.

Whilst the majority of tinnitus treatments involve sound and this require the ability to hear those sounds, others under investigation may be suitable for trialing in Deaf adults although do not. E.g. An open trial of Auditory Brain Implant (NCT02630589) excludes those with PTA above 90 dB in the ipsilateral ear, and another open trail on laser light therapy (NCT05374421) excludes anyone with age-related hearing loss or conductive hearing loss. Rationale for exclusions based in hearing loss should be well articulated in trial reports. Beyond any issues with access to sound it is likely that many researchers exclude participants who have severe-to-profound hearing loss in an effort to reduce the number of potential confounders and have a more homogenous study population. Problems with tinnitus may be conflated with those caused by hearing loss (56) so this effect may be more pronounced in those with more severe hearing loss.

### Cochlear implant studies where tinnitus was a secondary outcome

Unilateral cochlear implant is recommended for hearing restoration in people with severe-to-profound hearing loss who do not benefit sufficiently from acoustic hearing aids (57, 58). Van de Heyning et al. (58) named few centers worldwide that have reported offering cochlear implants for the purpose of address an individual's tinnitus in addition to their profound hearing loss, but usually only under highly specific conditions or strict criteria. For example, a clinic in Belgium reported implanting patients who complained of tinnitus but only if their tinnitus was the result of a hearing loss, whereas a clinic in Austria reportedly implanted profoundly deaf patients not meeting the standard criteria if they expected to receive more benefits than just restoration of hearing. Hence, these records either studied overall tinnitus suppression following cochlear implantation or investigated specific mechanisms by which the cochlear implant acts to suppress the tinnitus such as electrode insertion or cochlear programming.

These included investigating the degree to which patient related factors such as age, or implant factors such as electrode insertion, programming, and duration of use of device can predicate outcome. Also, for patients receiving a cochlear implant, their residual hearing at 250 Hz can be a positively predictive factor for tinnitus suppression post implantation, which could be beneficial in patients counseling (37).

Finally, few records looked at time as a factor for tinnitus suppression after cochlear implantation, especially in those patients who received cochlear implant but continued to complain of bothersome tinnitus, thus, received a second sequential implant for their tinnitus (46). Although, tinnitus improved in these studies it is unclear whether this was due to the person developing coping strategies or was part of an adaptation mechanism in the auditory brain due to auditory activation following cochlear implantation.

Tinnitus counseling is an important factor in tinnitus management which is lacking in these studies as well as the need for Deaf and severe-to-profoundly hearing-impaired adults to receive personalized management. A cochlear implant is a feasible method of providing hearing restoration but has also been demonstrated to have some efficacy in tinnitus suppression, although the results are variable and cases of tinnitus induction by cochlear implants were also reported. Hence, tinnitus patients should receive vigorous counseling sessions and must engage in the treatment plan.

Research describing advancements in tinnitus management in deaf populations is greatly needed due to significant increased numbers of cases of severe and profound hearing impairments in combination with improved overall life expectancy. All included studies used one treatment method, however due to the heterogeneity of tinnitus pathophysiology and different personal experiences, researchers are looking more into combination of treatments such as sound therapy, personalized counseling, hearing aids, and CBT. A recent multicentre clinical trial involved a comparison of the effects of the single and combination therapy, i.e., hearing aids alone or hearing aids and cognitive behavioral therapy, or hearing aids and structured counseling or hearing aids and sound therapy (59). However, it excluded participants with severe hearing loss due to barriers in communication, which again demonstrates the need for research into adapting the existent or developing new management methods for those who are Deaf or have severe-to-profound hearing loss.

### Authors' recommendations

This review highlights the lack of dedicated research involving adults who have severe-to-profound hearing loss. Researchers should clearly justify excluding this population form their tinnitus studies, and where it is not justified, should ensure studies are adequately resourced to be inclusive, and statistical analysis plans adequately consider hearing loss as a potential confounder. To be confident of outcomes it is also important to adequately screen and disambiguate the problems due to tinnitus from those due to hearing loss, e.g., using the Tinnitus and Hearing Survey (56). We recommend greater involvement of carers or significant others, the provision of sign interpreters, and the use of accessible media such as text over audio to include D/deaf adults in tinnitus research. Deaf adults should be involved in setting the research agenda, informing study design, and promoting participation, to ensure inclusivity is maximized. Some recommendations for tinnitus research in D/deaf populations have been identified in clinical practice guidelines, e.g., NICE (5) recommends research to (1) identify the most clinically and cost-effective tinnitus questionnaire to assess tinnitus in people who are d/Deaf, (2) evaluate clinical and cost effectiveness of amplification devices for people who are d/Deaf, and (3) evaluate clinical and cost effectiveness of psychological therapies for people who are d/Deaf and have tinnitus-related distress. There will require multiple approaches to evaluate existing, modified, or newly developed tools and treatments. Before these questions can be addressed, we recommend qualitative enquiry is needed to understand the lived experience of tinnitus more fully in d/Deaf adults. This could inform large scale quantitative enquiry (e.g., online survey) to understand the breadth and scale of tinnitus problems in d/Deaf adults. A formal prioritization exercise involving d/Deaf adults with tinnitus and clinicians with expertise in deafness and/or tinnitus would elevate the profile of research in this area, as has been done with success for other topic areas within the field of hearing (60, 61).

### Limitations

Because of resource limitations this review only examined studies available in English. The findings may therefore not generalize to other populations and their experiences where there may be a significant literature published in other languages. For the same reason it is likely that not all interventions that have been trialed for tinnitus in D/deaf adults have been captured. The review was also limited to studies reporting adult populations, so findings and recommendations cannot be applied to d/Deaf child populations.

## Conclusion

This scoping review aimed to catalog the experience of, assessment, and treatment of tinnitus in adults who are Deaf or have profound hearing loss. It is evident that there is very limited research in this area. Although this review included many records, most focused on the provision of cochlear implants for severe-to-profound hearing loss, with assessment and measurement of tinnitus as a baseline or secondary outcome. Largely missing in the literature are empirical studies that seek primarily to understand the nature of the experience of tinnitus in people with no or little residual hearing.

## Data availability statement

The original contributions presented in the study are included in the article/supplementary material, further inquiries can be directed to the corresponding author/s.

## Author contributions

LA performed literature searches, selected records for inclusion, extracted. DH and MS screened records and extracted data. DH, MS, and LA interpreted the data and drafted the manuscript. CC, HH, and RD provided critical feedback as part of scoping review stage six, and contribute to the final interpretation and content of the manuscript. All authors contributed to the article and approved the submitted version.

## Funding

LA is funded by King Abdul-Aziz University Hospital (KAU), Jeddah, Kingdom of Saudi Arabia. DH and MS are funded through the National Institute for Health and Care Research (NIHR) Biomedical Research Centre programme. The views expressed are those of the authors and not necessarily those of the NIHR, the NHS, the Department of Health, and Social Care. CC is funded by UNICAMP University of Campinas, Brazil, HH is funded by ENT Department, Hospital Cuf Tejo—Nova Medical School, Lisbon, Portugal and finally RD is funded by Sir Peter Mansfield Imaging Centre, School of Physics and Astronomy, University of Nottingham, United Kingdom. The funders had no role in preparation of the manuscript.

## Conflict of interest

The authors declare that the research was conducted in the absence of any commercial or financial relationships that could be construed as a potential conflict of interest.

## Publisher's note

All claims expressed in this article are solely those of the authors and do not necessarily represent those of their affiliated organizations, or those of the publisher, the editors and the reviewers. Any product that may be evaluated in this article, or claim that may be made by its manufacturer, is not guaranteed or endorsed by the publisher.
